# Fuzzy logic based on-line fault detection and classification in transmission line

**DOI:** 10.1186/s40064-016-2669-4

**Published:** 2016-07-07

**Authors:** Shuma Adhikari, Nidul Sinha, Thingam Dorendrajit

**Affiliations:** Department of Electrical Engineering, National Institute of Technology Manipur, Imphal, Manipur India; Department of Electrical Engineering, National Institute of Technology Silchar, Silchar, Assam India

**Keywords:** Fault, Power system protection, CRIO, Fuzzy logic

## Abstract

This study presents fuzzy logic based online fault detection and classification of transmission line using Programmable Automation and Control technology based National Instrument Compact Reconfigurable i/o (CRIO) devices. The LabVIEW software combined with CRIO can perform real time data acquisition of transmission line. When fault occurs in the system current waveforms are distorted due to transients and their pattern changes according to the type of fault in the system. The three phase alternating current, zero sequence and positive sequence current data generated by LabVIEW through CRIO-9067 are processed directly for relaying. The result shows that proposed technique is capable of right tripping action and classification of type of fault at high speed therefore can be employed in practical application.

## Background

Modern power system is a complex network and requires high-speed, precise, and reliable protective system. Faults in power system are unavoidable and overhead transmission line faults are generally higher compare to other major components. Due to recent technology advances, new and improved devices for protection of power system are being designed and developed. Fault classification in double circuit line with conventional techniques is difficult due to mutual coupling between the two circuits (Jain 2013). This mutual coupling is compensated by taking zero sequence current into account. The goal of system protection includes detection, classification and identification of fault with minimum time delay. In order ensure stability and continuity of service, the faulty area of the circuit should be isolated without time delay. Various methods of fault detection, classification and isolation have been reported in literature. The most commonly used techniques for fault classification are: (1) artificial neural networks (Dalstein and Kuliche 1995; Song et al. 1996; Osman et al. 2005; Mahanty and Gupta 2004; Yadav and Dash 2014; Jamil et al. 2015) (2) wavelet transform techniques (Youssef 2003; Chanda et al. 2005; Liang et al. 1998; Zhao et al. 2000; Eristi 2013; Yadav and Swetapadma 2015a; Saber et al. 2015; Koley et al. 2015) (3) fuzzy and neuro-fuzzy techniques (Nguyen and Liao 2010; Mahanty and Gupta 2006). The neural network approach for fault classification is established as a successful methodology but it requires tedious training effort, hence it is time consuming and adds to the computation complexity. Similarly wavelet transform techniques are computationally complex. The fuzzy logic based fault classification techniques are comparatively simpler as it requires only some linguistic rules. In (Ferrero et al. 1995) identified the nature of fault (whether LG or LLG), but the involved phases in the fault could not been identified and phase fault is not considered. In (Wang and Keerthipala 1998) reported the improved technique based on fuzzy-neural approach and considered both the symmetrical and unsymmetrical fault. But this method required extra effort to obtain training of ANN. In (Dash et al. 2000) showed all the ten types of fault identification by fuzzy-neural approach. In (Das and Reddy 2005; Yadav and Swetapadma 2015b; Saradarzadeh and Sanaye-Pasand 2014) proposed fuzzy logic methodology to identify the ten types of faults.

In this paper fuzzy logic based fault detection and classification on real time has been proposed. Post-fault three phase currents; zero sequence and positive sequence current samples are taken into account for fault classification. The proposed logic detects and classifies the faults at maximum delay of 100 ms or less with higher accuracy and also this speed can be further increased and detection time can be improved. Real time data acquisition ensures control within specified time limit.

## Methods

**Fig. 1:**
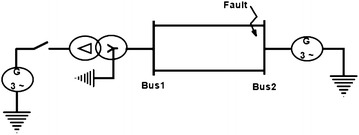
Single line diagram of two bus system

The method adopted for the study is applied on single line diagram shown in Fig. 1. 20 numbers of different faults have been created on test-bed for tuning the fuzzy membership function and fuzzy rules. The Data are acquired through CRIO and post fault data generated for different types of fault are used to evaluate the performance of the proposed fuzzy logic based fault classification system. The power system is developed taking into consideration the acquired data as shown in Fig. 1 in Lab view software. The fuzzy logic based fault classification is first experimented i.e. on offline environment for finding the optimal system. This optimal fuzzy logic based classification system is then applied on the system for any fault on real time. It is observed during the analysis of the data that depending on the type of fault i.e. line to ground faults, line to line faults, line to line to ground faults or three phases fault, the waveform changes accordingly. It is significant to mention that during fault the voltage tends to reduce to zero and current tends to rise.

Different types of faults are characterized in terms of δ_1_, δ_2_, δ_3_ and δ_4_, which calculations are shown below (Susilo et al. 2013). Post fault current samples are solved as below.

$$\delta_{1} = \frac{{{\text{I}}_{\text{a}} - {\text{I}}_{\text{b}} }}{{\hbox{max} \left( {I_{a} ,I_{b} ,I_{c} } \right)}}$$

$$\delta_{2} = \frac{{{\text{I}}_{\text{b}} - {\text{I}}_{\text{c}} }}{{{ \hbox{max} }\left( {I_{a} ,I_{b} ,I_{c} } \right)}}$$

$$\delta_{3} = \frac{{{\text{I}}_{\text{c}} - {\text{I}}_{\text{a}} }}{{{ \hbox{max} }\left( {I_{a} ,I_{b} ,I_{c} } \right)}}$$

$$\delta_{4} = \frac{{{\text{I}}_{0} }}{{{\text{I}}_{1} }}$$

where I_a_, I_b_, and I_c_ represent the sample of three phase currents. I_0_ and I_1_ are zero sequence and positive sequence current. Fuzzy rule based method for fault classification is developed on the basis of δ_1_, δ_2_, δ_3_, δ_4_. Zero sequence current, I_0_ has been taken into account to detect the presence of ground fault and δ_4_ represents the ground fault detection.

**Fig. 2:**
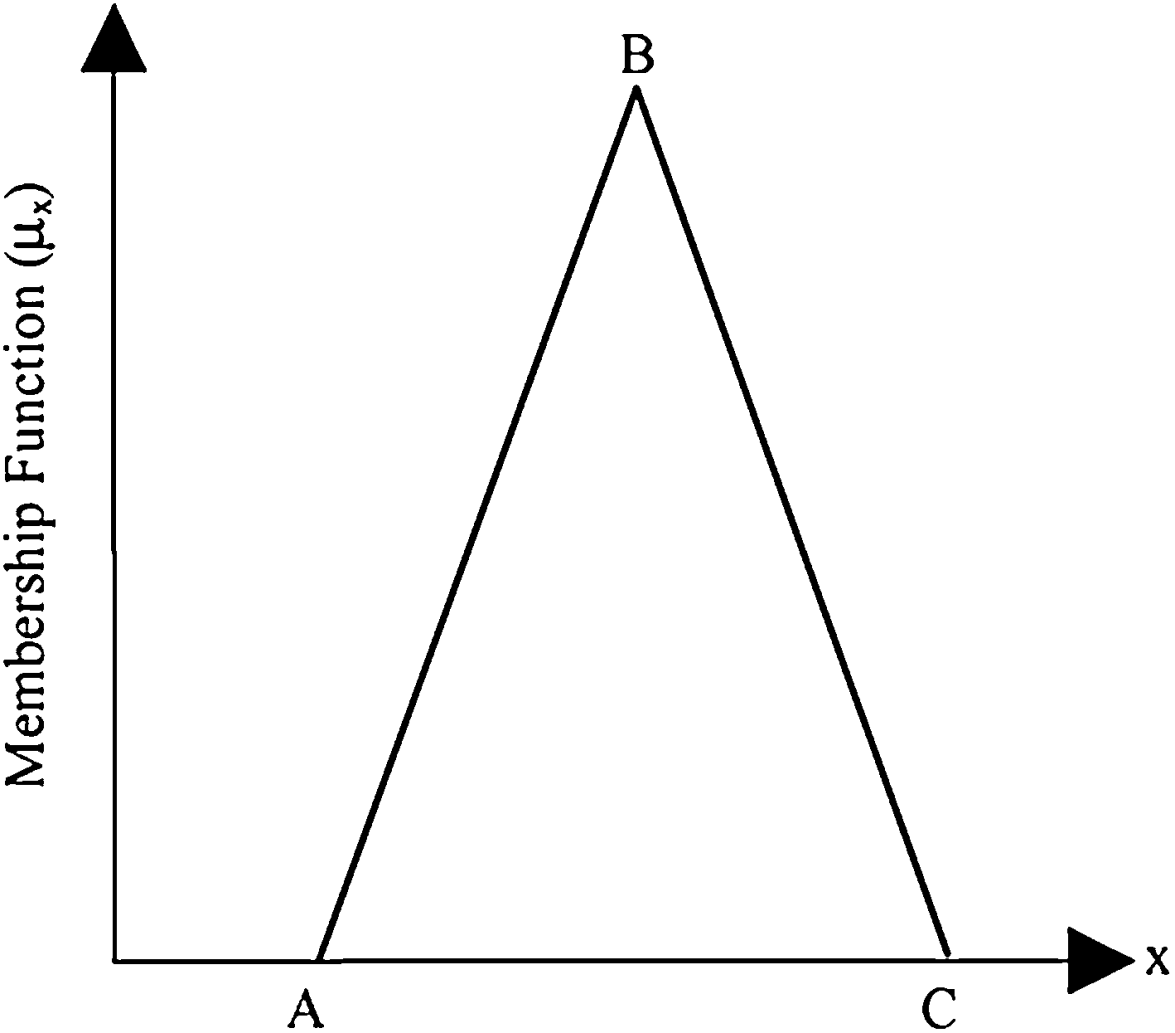
Triangular fuzzy membership function

Fuzzy rule base for fault classification:

- If δ_1_ is high and δ_2_ is medium and δ_3_ is low and δ_4_ is high it is an L_a_ − G fault;
- If δ_1_ is low and δ_2_ is high and δ_3_ is medium and δ_4_ is high it is an L_b_ − G fault;
- If δ_1_ is medium and δ_2_ is low and δ_3_ is high and δ_4_ is high it is an L_c_ − G fault;
- If δ_1_ is medium and δ_2_ is high and δ_3_ is low and δ_4_ is low it is an L_a_ − L_b_ fault;
- If δ_1_ is low and δ_2_ is medium and δ_3_ is high and δ_4_ is low it is an L_a_ − L_c_ fault;
- If δ_1_ is high and δ_2_ is low and δ_3_ is medium and δ_4_ is low it is an L_b_ − L_c_ fault;
- If δ_1_ is medium and δ_2_ is high and δ_3_ is low and δ_4_ is high it is an L_a_ − L_b_ − G fault;
- If δ_1_ is low and δ_2_ is medium and δ_3_ is high and δ_4_ is high it is an L_a_ − L_c_ − G fault;
- If δ_1_ is high and δ_2_ is low and δ_3_ is medium and δ_4_ is high it is an L_b_ − L_c_ − G fault;
- If δ_1_ is medium and δ_2_ is medium and δ_3_ is medium and δ_4_ is low it is an L_a_ − L_b_ − L_c_ fault;

The triangular membership function has been used to present different fuzzy variables in the antecedent and consequent parts of the fuzzy rules as shown in Fig. 2. Mendal (1995) describes the triangular membership function as triplets with respect to the points A, B and C. It is observed that points A and C have membership value of 0.0 while point B has membership value of 1.0. Extensive study has been carried out to select proper triplets values of triangular membership function of δ_1_, δ_2_, δ_3_ and δ_4_. The selected triplets for triangular membership function of fuzzy variables in antecedents parts and consequent part are shown in Tables 1, 2 and 3 respectively.

**Table 1:** Fuzzy variables in the antecedent parts of fuzzy rules for δ_1_, δ_2_, δ_3_

Input variables	Triangular triplets		
	A	B	C
Low	−1	1	−0.56
Medium	−0.61	1	0.61
High	0.54	1	1

**Table 2:** Fuzzy variables in the antecedent parts of fuzzy rules for δ_4_

Input variables	Triangular triplets		
	A	B	C
Low	−2	1	0.1
High	−0.01	1	3

**Table 3:** Fuzzy variables in the consequent parts of fuzzy rules

Fuzzy variables	Triplets		
	A	B	C
L_a_ − G	6.5	7	7.5
L_b_ − G	4.5	5	5.5
L_c_ − G	2.5	3	3.5
L_a_ − L_b_	63.5	64	64.5
L_a_ − L_c_	60.5	61	61.5
L_b_ − L_c_	65.5	66	66.5
L_a_ − L_b_ − G	76.5	77	77.5
L_a_ − L_c_ − G	94.5	95	95.5
L_b_ − L_c_ − G	96.5	97	97.5
L_a_ − L_b_ − L_c_	73.5	74	74.5

### Block diagram of fuzzy logic based fault classification

**Fig. 3:**
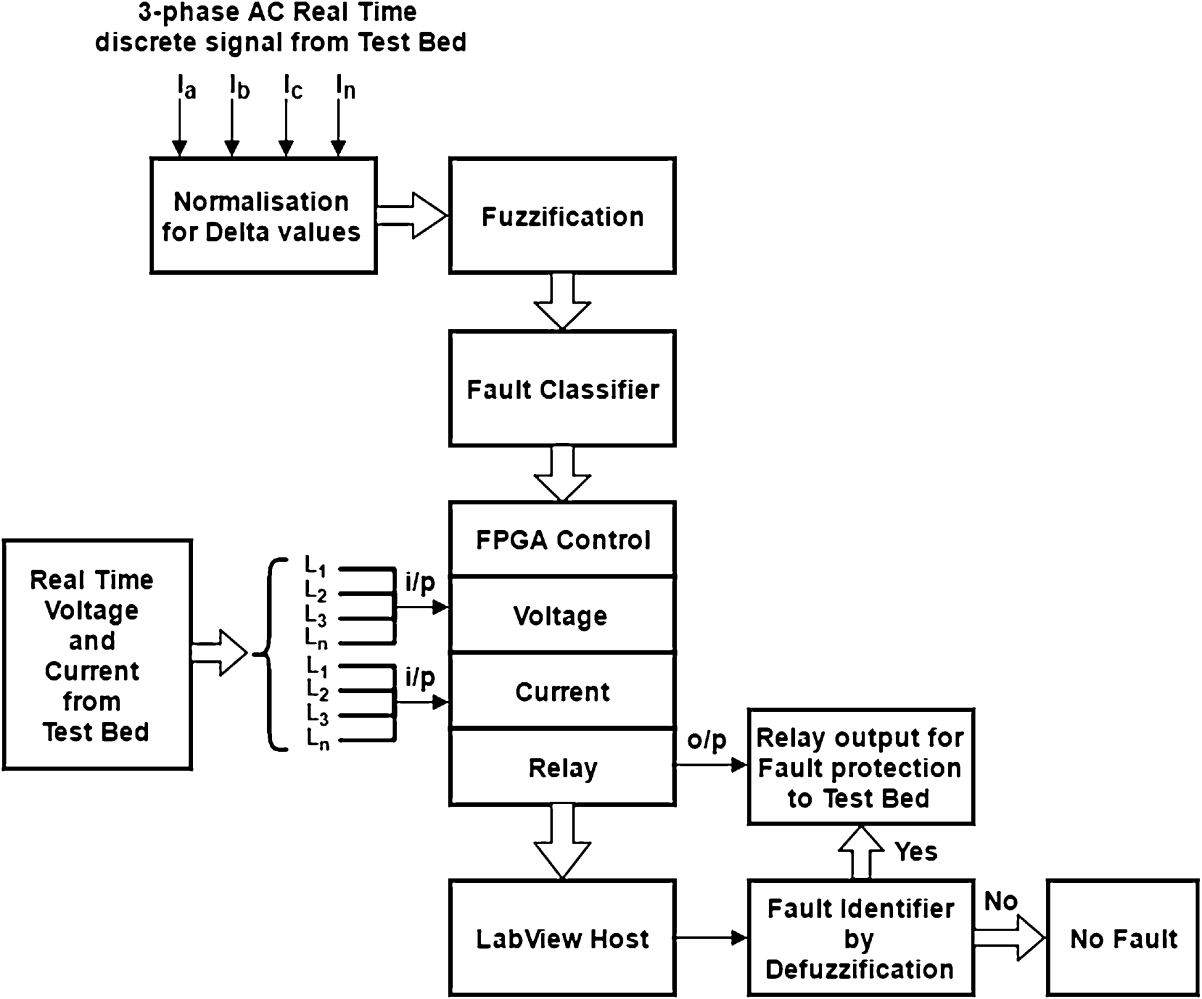
Block diagram of the proposed methodology

Figure 3 presents the block diagram of the proposed methodology. The ADC (analog to digital converter) is connected with FPGA (field-programmable gate array) hardware. The FPGA can directly access the ADC acquired values and send it to RT (real time) processor. The FPGA uses a 40 M Hz clock for its operation. The Sampling rate of FPGA is set at 10 kS/s which give 200 samples for each cycle. For every sample the FPGA acquire value is put into a FIFO (fast in fast out) queue, which can be accessed from the RT processor. The RT processor polls 1000 values for each channel, which means 5 consecutive cycles of 50 Hz signal. Once 5 cycle data is present, the RT processes the data and measures the RMS value of the signal acquired, and also checks for fault conditions. The sampling rate and the number of sample to detect the fault can be varied using the user control; this gives an option to change the parameters of the fault detector leading to improvement of efficiency, accuracy and response time. Three phase current data from test-bed have been acquired through CRIO. The signals acquired are normalized and different faults are characterized in terms of δ_1_, δ_2_, δ_3_ and δ_4_. After analysis of data obtained, triplet values are selected in antecedent and consequent parts to represent various fuzzy variables. Rules base are then prepared for classifying the fault type. After successful compilation of simulation fuzzy logic, the generated logic is dumped to the Field Programmable Gate array (FPGA) control LabVIEW hardware. In CRIO three modules have been used for high voltage data acquisition modules, high current data acquisition module and relay switch module for protection. The real time, line voltage and current values from the test bed are used as input to the FPGA control. When fault occurs in the system, relay switch module detect fault and the relay will trip after intentional 5 ms delay. The type of fault occurring in the system will be displayed on the host PC.

### Real time monitoring and controlling

**Fig. 4:**
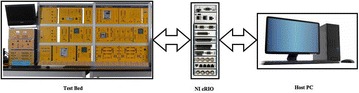
Programmable automation and control technology of test-bed

**Fig. 5:**
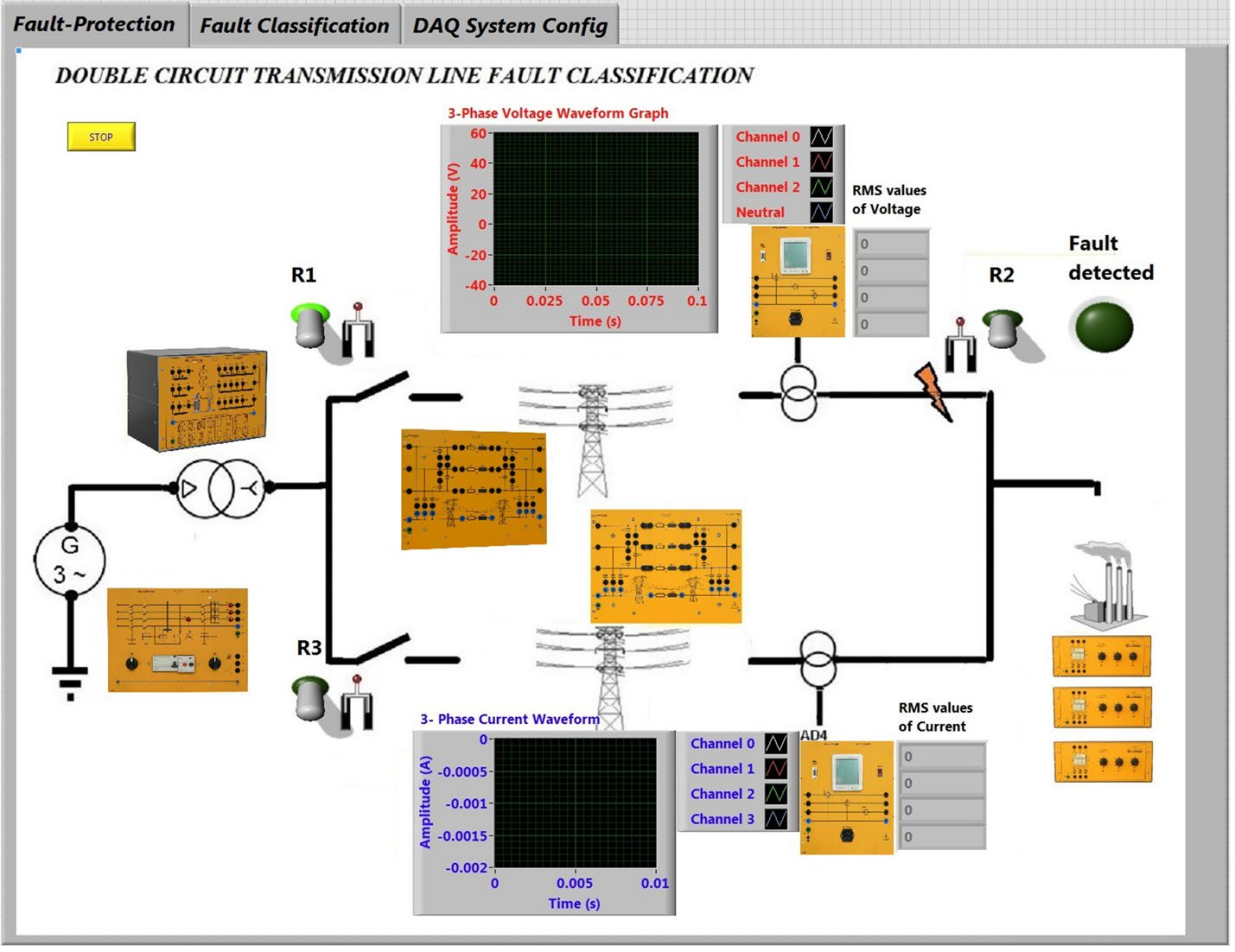
Front panel of the LabvIEW GUI

**Fig. 6:**
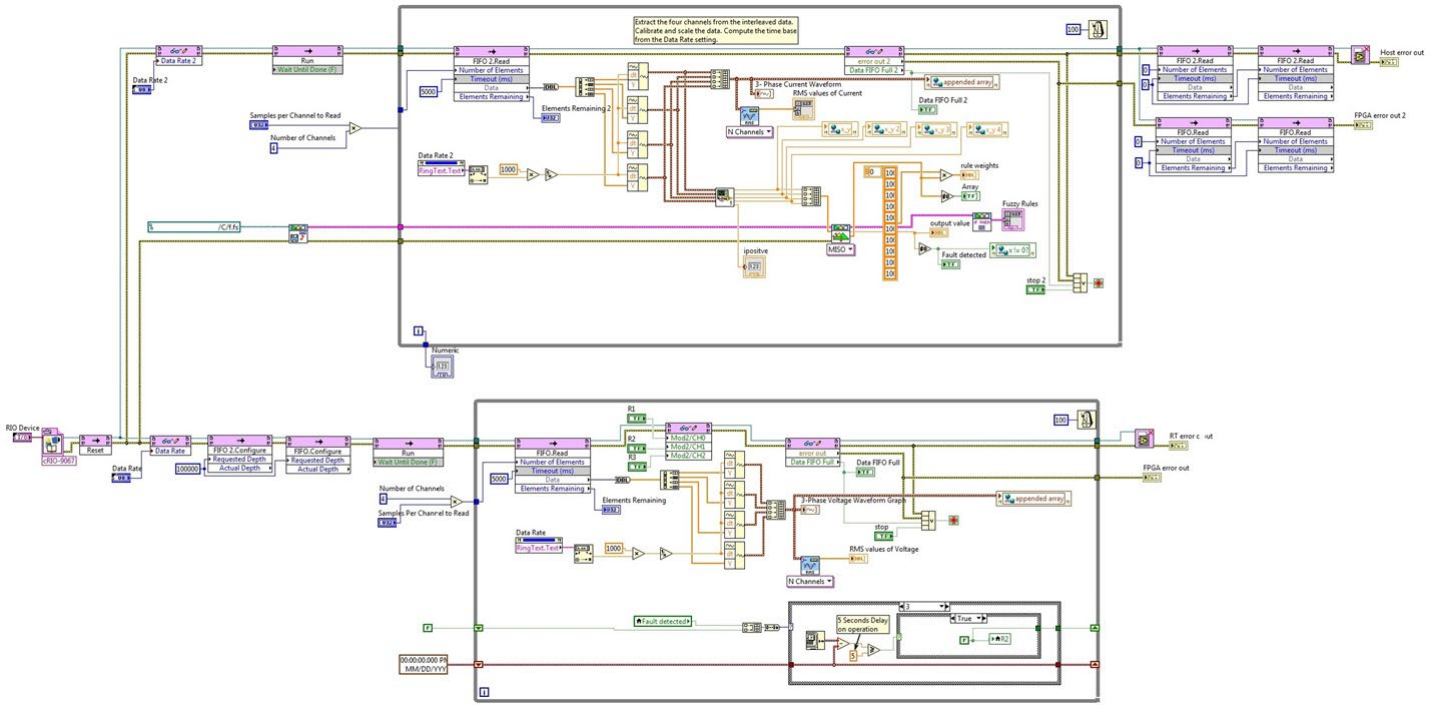
The program block diagram of fault classification

Laboratory Virtual Instrument Engineering Workbench (LabVIEW) is a powerful and flexible instrumentation and analysis software application tool which was developed in 1986 by the National Instruments. LabVIEW is extremely flexible and commonly used for data acquisition, instrument control, data processing and industrial automation. The CRIO device can interface between computers and Test-bed set up. Figure 4 shows schematic of real time monitoring and controlling. The LabVIEW 14.01 and CRIO 9067 systems have been implemented as SCADA to monitor real time parameters of test-bed. CRIO 9067 combines a dual-core processor, a reconfigurable FPGA, and eight slots for C Series I/O modules within one chassis. The CRIO systems consist of a real time processor running Real Time Operating System (RTOS) and also a FPGA backplane for accessing the I/O s. The RTOS and FPGA work mutually to ensure real time performance. C series I/O modules used for the study are—NI 9244, NI 9227, NI 9472, NI 9482, NI 9871, NI 9467. The NI 9244 module has 3 single-ended channels, 50 kS/s per channel simultaneous sample rate with 400 Vrms L-N, 800 Vrms L-L measurement range & 24-bit resolution and NI 9227 C Series current input module was designed to measure 5 A rms nominal and up to 14 A peak on each channel with channel-to-channel isolation with 50 kS/s/ch simultaneous sample rate. The NI 9871 has 4-Port, RS485/RS422 Serial Interface Module and used for RS485 data monitor on MODBUS. NI 9871 has baud rates up to 3.684 Mbaud per port. We can pass up to 1.28 Mbit/s of data between the module and CompactRIO. This ensure that the data monitored by the current and voltage monitor modules are correct and matching with the other measuring systems output. The NI 9467 provides GPS location information for measuring phasor values precisely. The digital output module and relay control module is used for protection of the grid. NI 9482 relay module is used. NI 9482 is a 4-channel, single-pole single throw (SPST) sourcing digital output module for NI Compact DAQ and CompactRIO. Each channel provides an SPST relay for switching signals up to 30 VDC (1.5 A), 60 VDC (1 A), 250 VAC (1.5 A) when all channels are being used. Figure 5 presents front panel of LabVIEW graphical user interface (GUI) created. Figure 6 shows real time program for fuzzy logic based fault classification.

### Hardware implementation

We have used National Instruments Controller with 667 MHz Dual-Core ARM Cortex-A9 processor running in the NI Linux Real-Time, also integrated Chassis has Artix-7 FPGA. LabVIEW 14 Version has been used for programming and implementation of logics. For the compilation process LabVIEW uses Xilinx Vivado 2013.4 as Compilation Tool. Table 4 shows the compilation result.

**Table 4:** Logic utilization table

Sl. no	Device utilization	Used	Available	Percentage of utilization
1	Total slices	4371	13,300	32.8
2	Slice register	11,038	106,400	10.4
3	Slice LUT’s	11,319	53,200	21.3
4	Block RAMs	24	140	17.1
5	DSP48s	2	220	0.9

**Fig. 7:**
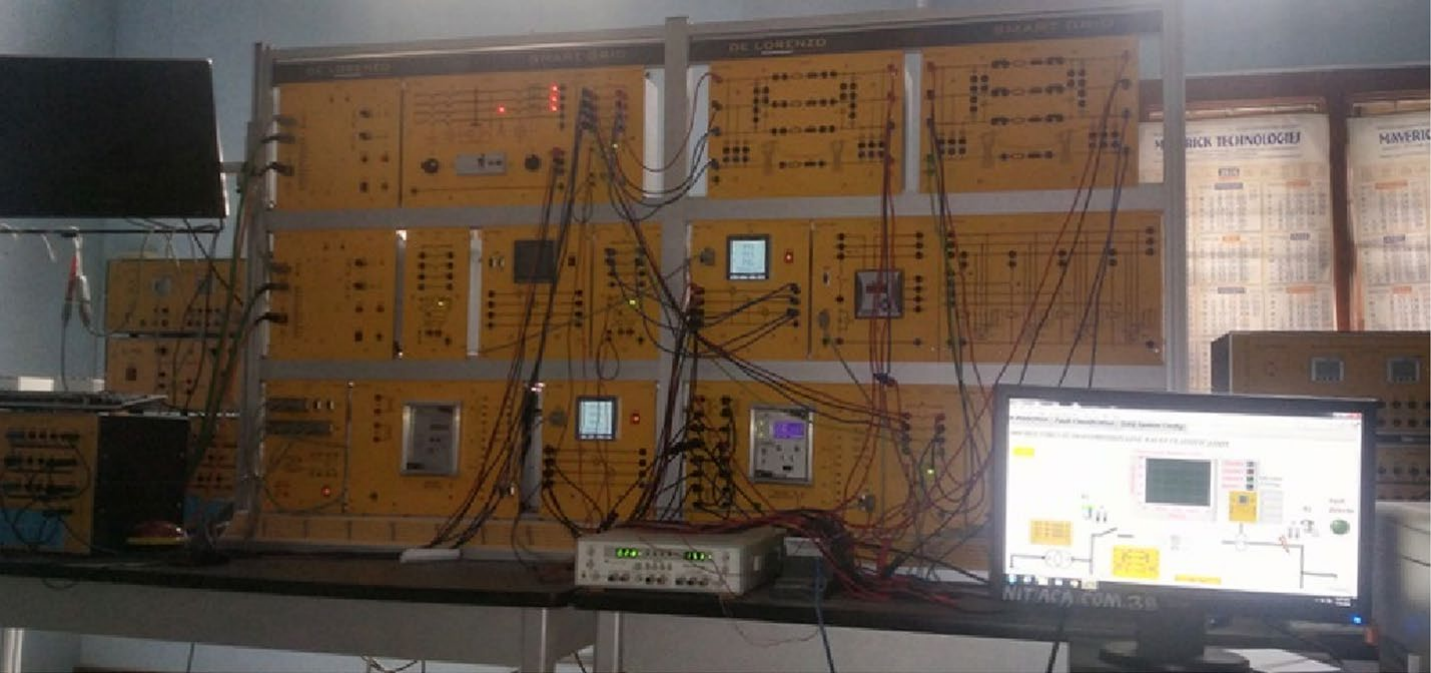
Picture of hardware setup

The proposed logic for fault detection and classification has been tested on an experimental transmission line module on 360 km of π-model. The picture of the hardware set-up is shown in the Fig. 7. Table 5 represent the data of the test-bed.

**Table 5:** Data of the test-bed

*Scale factor*	
Voltage	380 V:380 kV = 1:1000
Current	1 A:1000 A = 1:1000
*Line parameters*	
Resistance	R_L_ = 13 Ω
Inductance	L_L_ = 290 mH
Mutual capacitance	C_L_ = 0.5 µF
Earth capacitance	C_E_ = 1 µF
*Earth return parameter*	
Resistance	R_E_ = 11 Ω
Inductance	L_E_ = 250 mH
*Natural load*	
600 MW	
*Characteristic impedance*	
340 Ω	

## Results and discussion

**Fig. 8:**
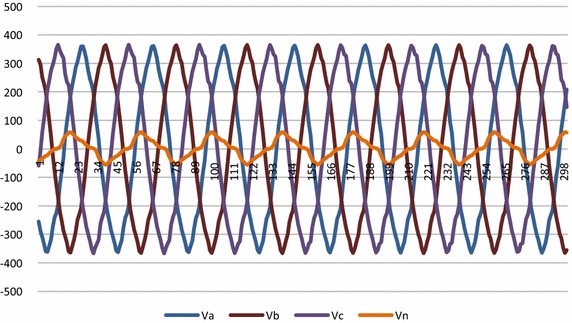
Voltage waveform of the system before fault

The hardware set up is connected properly. Ten different faults are created as shown in Fig. 1 on 360 km double circuit line. For fault detection and classification LabVIEW fuzzy logic tool kit has been used and the system is protected at 5 ms delay triggered using LabVIEW through CRIO controller relay module. It is worth to mention that proposed logic is able to detect fault, trip the line as well as classify type of the fault occurred. Figure 8 shows the voltage waveform before introduction of fault in the system.

**Fig. 9:**
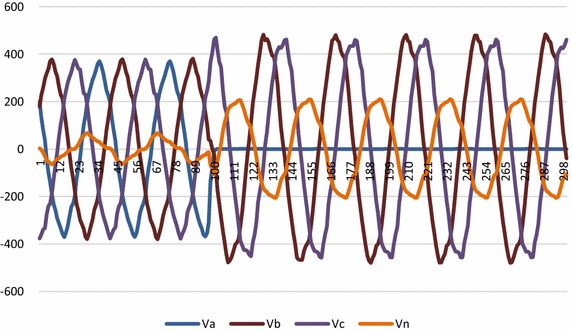
Voltage waveform for L_a_ − G fault

**Fig. 10:**
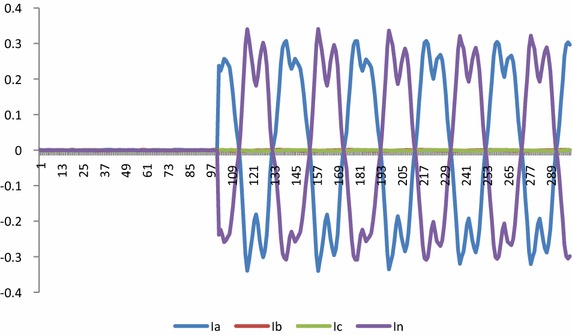
Current Waveform for L_a_ − G fault

**Fig. 11:**
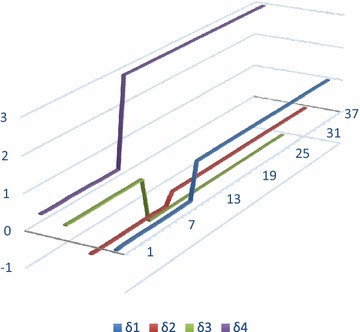
Output for δ_1_, δ_2_, δ_3_ and δ_4_ for fault classification during L_a_ − G fault

The voltage and current waveforms for La − G fault are presented in Figs. 9 and 10. It is observed that when fault occurs in the system, voltage of L_a_ reduces and current increases. Figure 11 presents graphical result of fault classification which follow fuzzy rule base of L_a_ − G fault i.e. δ_1_ is high and δ_2_ is medium and δ_3_ is low and δ_4_ is high.

**Fig. 12:**
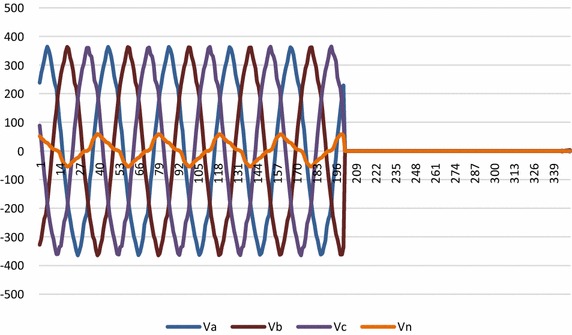
Voltage waveform for L_a_ − L_b_ − L_c_ fault

**Fig. 13:**
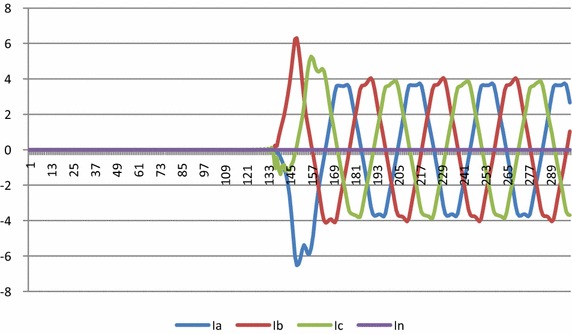
Current waveform for L_a_ − L_b_ − L_c_ fault

**Fig. 14:**
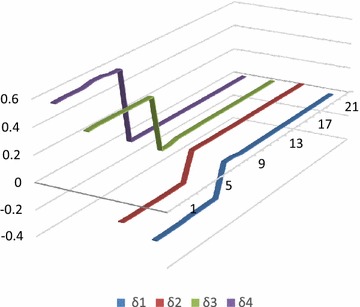
Output for δ_1_, δ_2_, δ_3_ and δ_4_ for fault classification during L_a_ − L_b_ − L_c_ fault

Figures 12 and 13 show voltage and current waveforms for L_a_ − L_b_ − L_c_ fault. It can be seen that when fault occurs in the system, voltage of L_a_, L_b_ and L_c_ reduces and current increases. It is also observed in Fig. 14 that δ1, δ_2_, δ_3_ and δ_4_ satisfy _fuzzy_ rule base for L_a_ − L_b_ − L_c_ fault.

The fuzzy logic based fault identification and classification is easy and simple since it only require computation of some ratios and differences of ratios of post fault current samples. When different fault are introduced in the system the corresponding fuzzy logic output are presented in Table 6. All the faults are checked graphically to confirm the accuracy of the proposed scheme. The proposed logic detects and classifies the fault accurately. The results show that the proposed logic is efficient and appropriate.

**Table 6:** Result of fuzzy logic based fault classification

Fault type	FLS input (δ_1_, δ_2_, δ_3_, δ_4_)	Fault currents (Ia, Ib, Ic, In)	FLS output
L_a_ − G	0.98737, −0.00296, −0.98441, 2.92775	−0.13610, −0.00158, −0.00073, 0.13671	6.85
L_b_ − G	−0.99090, 0.98533, 0.00556, 2.94215	0.00109, 0.27380, −0.00024, −0.27600	5.15
L_c_ − G	−0.00264, −0.98845, 0.99110, 2.95408	0.00012, −0.00036, −0.06103, 0.03784	3.30
L_a_ − L_b_	0.00017, 0.99949, −0.99951, 0.00034	3.05688, −3.05761, −0.00134, −0.00048	64.10
L_a_ − L_c_	0.99939, −0.99955, 0.00015, 0.00036	3.19409, −0.00024, −2.77185, −0.00073	66.15
L_b_ − L_c_	−0.99968, 0.00012, 0.99967, 0.00034	0.00024, −2.58276, 2.58154, −0.00036	61.30
L_a_ − L_b_ − G	0.01731, 0.98115, −0.99847, 0.07258	1.11511, −1.23242, −0.00231, 0.11669	77.10
L_a_ − L_c_ − G	0.98100, −0.99869, 0.01769, 0.09245	3.63757, −0.00085, −3.66918, 0.02856	95.40
L_b_ − L_c_ − G	−0.99946, 0.01475, 0.98471, 0.11095	0.00158, 1.94299, −1.63598, −0.31176	97.10
L_a_ − L_b_ − L_c_	0.00144, 0.00508, −0.00653, 0.00058	3.68237, −1.95690, −1.72717, −0.00036	74.25

## Conclusion

An approach for fault detection and classification for transmission line based on fuzzy logic is found to be very efficient and effective under different fault conditions. This technique can determine not only fault detection and fault classification but can also give automatic protection in real time. The system operation is fast, reliable, and secure. Proposed logic is simple since it requires only some linguistic rules. The results show that proposed techniques is simple, fast, reliable and secure.
